# Diversity and development of Indigenous rehabilitation professional student identity

**DOI:** 10.1186/s12909-024-05576-y

**Published:** 2024-05-30

**Authors:** Cara L. Brown, Debra Beach Ducharme, Kimberly Hart, Nichol Marsch, Louise Chartrand, Melissa Campbell, Danielle Peebles, Gayle Restall, Moni Fricke, Dustin Murdock, Jacquie Ripat

**Affiliations:** 1 Department of Occupational Therapy, College of Rehabilitation Sciences, Rady Faculty of Health Sciences, University of Manitoba, R106 - 771 McDermot Avenue, Winnipeg, Manitoba, R3E 0T6 Canada; 2 Ongomiizwin Indigenous Institute of Health and Healing - Education, Rady Faculty of Health Sciences, University of Manitoba, Winnipeg, Manitoba, Canada; 3 Department of Respiratory Therapy, College of Rehabilitation Sciences, Rady Faculty of Health Sciences, University of Manitoba, Winnipeg, Manitoba, Canada; 4 Independent Contractor, Winnipeg, Manitoba, Canada; 5 Department of Physical Therapy, College of Rehabilitation Sciences, Rady Faculty of Health Sciences, Winnipeg, Manitoba, Canada; 6 Adapt Physical Therapy Winnipeg, Winnipeg, Manitoba, Canada

**Keywords:** Rehabilitation, Student, Indigenous, Identity, Reconciliation, Post-secondary education, Health professional, Occupational therapy, Physical therapy, Respiratory therapy

## Abstract

**Background:**

In Canada, disparities between Indigenous and non-Indigenous Peoples continue to exist in health and education because of the past and current harms of racism and colonization. One step towards closing health gaps is clinicians who can provide health and social care services that are free of racism and mistrust. Indigenous health providers are in the best position to provide this culturally relevant and safe care to their own communities. Therefore, more Indigenous students graduating from health professional programs are required to meet these needs. Indigenous identity support can be a facilitator for Indigenous student academic success but developing one’s Indigenous identity can be challenging in post-secondary education environments. We explored how Indigenous rehabilitation students expressed, and wanted to be supported in their identity and academic success.

**Methods:**

Using a narrative inquiry approach, we conducted interviews with seven students from the occupational, physical, and respiratory therapy programs of a Canadian university. Students were asked to tell their story of learning about, applying to, and being in their rehabilitation program and how their Indigenous identity impacted these experiences. Data analysis was conducted by Indigenous and non-Indigenous team members, analyzing the stories on interaction of the participant with (1) themselves and others, (2) time, and (3) situation or place.

**Results:**

The researchers developed seven mini-stories, one for each participant, to illustrate the variation between participant experiences in the development of their Indigenous and professional identity, before and during their rehabilitation program. The students appreciated the opportunities afforded to them by being admitted to their programs in a Indigenous Peoples category, including identity affirmation. However, for most students, being in this category came with feared and/or experienced stigma. The work to develop a health professional identity brought even more complexity to the already complex work of developing and maintaining an Indigenous identity in the colonized university environment.

**Conclusion:**

This study highlights the complexity of developing a rehabilitation professional identity as an Indigenous student. The participant stories call for universities to transform into an environment where Indigenous students can be fully accepted for their unique gifts and the identities given to them at birth.

**Supplementary Information:**

The online version contains supplementary material available at 10.1186/s12909-024-05576-y.

## Background

Our health care systems are currently rife with institutionalized and personally mediated racism towards Indigenous Peoples that negatively impact health outcomes [1–3]. One important step towards culturally safer care is to foster the development of clinicians who can provide health and social care services that are free of racism and mistrust, and who can honour self-determination and the use of holistic health approaches with Indigenous Peoples and communities [4]. Indigenous health providers are in the best position to provide this culturally relevant and safe care to their own communities [1, 5], and to develop health care that is grounded in Indigenous People’s cultures and perspectives [6]. However, not only are current graduation rates of Indigenous health professionals not adequate to meet these needs [7], embracing and building upon one’s own Indigenous identity is challenging for some students in the post-secondary education environment.

Around the world, there are fewer Indigenous students entering and completing university education compared to non-Indigenous students [8]. We know this is due to the impacts of colonization and has resulted in Indigenous health professional students (nursing, medical and health sciences) having more stressors in adapting to an unfamiliar learning environment than non-Indigenous students [7, 9, 10]. Factors negatively impacting retention and success include racism and discrimination in the classroom and in clinical rotations as well as numerous systemic barriers for non-completion of their degree. These barriers have been well-documented in the literature and include navigating Eurocentric systems, lack of adequate academic support, caring for family members while being a student, financial insecurity, less social support (particularly for students leaving their home communities to attend university), and deficit-based academic models [7, 9–11].

The literature has identified that cultural supports - i.e., strategies that support the cultural identity and expression of the individual - can increase student success rates [9]. Cultural supports include: Indigenous-specific philosophies and practices with Elders, Knowledge Keepers and counselors; a personal network of Indigenous and non-Indigenous mentors, faculty, and classmates; help with navigating financial supports; culturally appropriate recruitment and admission processes; pre-entry upstream recruitment programs; embedded Indigenous curriculum that centres Indigenous ways of knowing and being; flexible delivery of content; social and financial support; and program flexibility that allows students to return if they need to leave for some reason [9–12]. Anderson and colleagues [9] suggest that fostering, affirming and/or supporting a strong cultural identity is central to these supports, but expressing Indigenous identity in a colonized university environment can contribute to students experiencing further victimization [13, 14].

Indigenous scholars have described how identity is a deeply complex topic and experience for Indigenous Peoples. Weaver, a Lakota woman, explained that generations of Indigenous Peoples hid their identity to remain safe, or had their culture stripped from them because of colonization [15]. Some Indigenous Peoples left their own culture and practices, not because of a desire to lose this part of their identity, but rather to cope with racism and trauma [13]. This social and political history has resulted in some Indigenous Peoples being separated from their identity. Further, pervasive negative images and stereotypes of Indigenous Peoples that persist today means that some Indigenous Peoples do not embrace their identity because of fear of stigma. Fox [13], from the xʷməθkʷəy̓əm (Musqueam) First Nation, associates the legacy of colonization and racism towards Indigenous Peoples with the challenging experiences of Indigenous post-secondary students. She explained that while Indigenous identity has adapted over time and with new learning, it is linked “to ancestors, to languages and to cultural traditions” (p. 246). When these Indigenous ways of being and knowing are not represented in post-secondary institutions, students do not feel connected to the university (14).

Ensuring there are enough Indigenous health providers to provide culturally relevant and safe care for Indigenous Peoples is a responsible moral and ethical goal. This includes rehabilitation professionals who have received little attention in the literature to date regarding recruitment and retention to increase the number of Indigenous rehabilitation health professionals. Indigenous occupational therapy, physical therapy and respiratory therapy professionals are needed to support Indigenous Peoples who are contending with chronic disease and disability due to colonialism, racism and imposed poverty that has directly impacted health status and health care access [1, 5]. Indigenous Peoples have higher rates of injury, diabetes and diabetes -related complications [3, 16] that require rehabilitation services for restoring functional abilities.

Our initial goal for this project was to learn how to better support Indigenous rehabilitation students during their health professional programs, to develop program-specific goals for improving Indigenous recruitment, retention, and student experience. The identity of Indigenous participants, and how they wanted to be supported as Indigenous post-secondary health professional students was a main point of discussion in the study, and we present these findings in this paper. Indigenous authors [14, 17] have noted a lack of inclusion of Indigenous student voices in literature about academic success, and thus our work lies on these voices.

## Theoretical framework and methodology

We were guided by narrative inquiry, an approach that acknowledges the importance of storytelling [18, 19]. This methodology is commonly used for education research as it provides a voice for students and “places the emphasis on stories in all aspects of life (p.330)” [19]. In narrative inquiry, the researchers learn about the experiences of participants in a particular setting by hearing their stories. The story typically includes personal experiences, as well as social experiences and interactions of the individual. The stories are then analyzed and retold by the researchers. The retelling highlights the key elements of all the stories told by the participants, typically in chronological order [19].

Narrative inquiry has been used by other authors for creating a space for Indigenous Peoples to tell “critical stories of personal and professional experience” [20]. Storytelling is a powerful way to explore identity as it supports individuals to integrate their life experience with an “evolving story of the self (p.20)”; identity is formed through the narrative that the storyteller creates of their life [21]. Identity is a long-studied concept, with dominant theories including identity theory and social identity theory. Social identity has been defined as “that part of an individual’s self-concept which derives from his (sic.) knowledge of his (sic.) membership of a social group (or groups) together with the value and emotional significance attached to that membership (p.255)” [22]. These theories view identity as a reflexive process, where our own identity is defined by the group in which we belong according to social categories, and that each individual belongs to multiple social categories. Self-identification of a particular category shapes how an individual enacts their roles within society [23]. In the case of professional identity formation, students come to their professional program with an already established identity, and then are re-forming their identity to integrate the identity of ‘health professional’. While this basic concept of identity provides a helpful starting place to consider identity, our team did not use these theories explicitly to guide our study design and analysis. Recognizing that these theories originated from a Western worldview, we wanted to centre Indigenous identity viewpoints using Indigenous student and scholar voices. The narrative framework allowed us to create space for the Indigenous students in this study to share their identity narrative at a critical point of identity reformation and then reflect on their stories considering the writings of Indigenous scholars.

The generation and understanding of knowledge in research are directly influenced by the positionality of the researchers. In narrative inquiry, the researcher’s own contexts and the way they see themselves influences the re-telling of participant stories [24]. We are a group of non-Indigenous and Indigenous researchers, rehabilitation clinicians, and Indigenous student support faculty in the College of Rehabilitation Sciences at the University of Manitoba. Cara led this project. She is a white settler of Western European and Icelandic origins, an occupational therapist, a teacher and researcher. Debra is Anishinaabe and a member of Lake Manitoba First Nation. She has experience in elementary and secondary education as a teacher, counsellor, consultant, director and administrator. She now works to implement the Truth and Reconciliation Calls to Action at the post-secondary level, by promoting the revitalization of Indigenous worldviews, languages and culture. Kimberly is a proud member of the Fisher River Cree Nation, Manitoba and has a Master of Social Work degree. In appreciation for the support that she received as a post-secondary student from her family, Kimberly now has the honour of supporting Indigenous learners through their own academic journeys. Nichol Marsch is a Métis artist and school-based occupational therapist in rural and remote First Nation communities in Manitoba. She joined this project as a part of a research mentorship program for undergraduate students. Louise is a French white woman, first generation PhD (sociology), second generation health care professional. Louise recognizes herself as being privileged to be an assistant professor in respiratory therapy. She also recognizes herself to be neuro divergent, with dyslexia. Louise believed that the fluidity of the concept of identity was lacking especially when addressing Indigenous experiences in health care academic programs. Melissa Campbell is a Métis occupational therapist from Stonewall, Manitoba with family ties to the communities of St. Laurent and Teulon, Manitoba. She works in and with First Nation communities through the Jordan’s Principle - Child First Initiative and is a sessional instructor in the Department of Occupational Therapy at the University of Manitoba. Danielle is currently a social worker in the North End, inner city and downtown community of Winipikk (Winnipeg). She has ties to three First Nation communities in Treaty 1 and Treaty 2 territories, Sakgeeng First Nation, Keeseekowenin First Nation and Peguis First Nation. Gayle is a third-generation white setter of Eastern and Western European ancestry. She is an occupational therapist and scholar. Moni is an uninvited guest on Turtle Island who self identifies as a first-generation white settler of European ancestry. In her previous work as a clinician and researcher working in Indigenous communities, she is very cognizant of the need for more Indigenous rehabilitation therapists in the workforce; in her academic position, she is interested in how best to support Indigenous students. Dustin is a member of Fisher River Cree Nation, Manitoba and has family ties to Grand Rapids, Manitoba. He completed a master’s in physical therapy degree from the University of Manitoba in 2020. He has an interest in developing both capacity and awareness of the physical therapy profession in First Nations communities. Jacquie is a second-generation white settler of European ancestry. She is a university teacher and researcher in the occupational therapy program.

## Methods

### Setting

This study was conducted in a large Canadian university in the province with the highest proportion of Indigenous Peoples in the country at 17% of the population [25]. The university has Indigenous-specific support programs for undergraduate students including a program specifically for Indigenous students who are considering a career in health care, located on the main campus of the university (will be referred to in this paper as the Undergraduate Indigenous Health Career Program).

There are three rehabilitation programs located on the health campus, approximately five kilometers away from the main campus. At the time of data collection, there were 50 students admitted per year in the occupational therapy and physical therapy programs respectively, and 17 students admitted per year to the respiratory therapy program. The occupational and physical therapy programs are clinical master’s programs (two years in length), while the respiratory therapy program is a bachelor program (three years in length). To increase Indigenous representation in rehabilitation professions, all programs protect 20% of program seats for a Canadian Indigenous People’s category. There is an Indigenous Health Professional Student Support program specifically for the health professional programs. It is a place and space for prospective and currently enrolled First Nations, Métis and Inuit students in health professional programs to meet, study and explore careers and culture. This program provides a welcoming environment that assists students to meet their academic potential through a variety of culturally relevant programs, resources and supports.

### Participants

In the spring of 2019, we invited 45 self-identifying Indigenous students and recent graduates (graduating classes of 2018, 2019, and 2020) from the programs of occupational therapy, physical therapy, and respiratory therapy to participate in the study. Recruitment strategies included email, social media, classroom announcements, and announcements in newsletters for Indigenous students.

### Data collection and analysis

Students shared their stories through individual interviews conducted by an Indigenous registered social worker, who had experience conducting qualitative research and did not work in the educational program of the participants (DP). We felt these interviewer characteristics would increase the comfort of the students in sharing information freely and allow the interviewer to respond to any emotional distress if required. Students were given the option of having an Indigenous Elder present during the interview; no participants requested an Elder be present in the interview. The interviews were conducted within the Indigenous Health Professional Student Support program offices. The interviews ranged in length from 15 to 54 min, with an average interview length of 45 min.

The students were asked to share their personal story of how they decided to enter their chosen health professional program, and their experience in applying for, and attending the program. The interview started with the broad open-ended question: “What is your story of how you learned about (occupational therapy, physical therapy or respiratory therapy), and your experience with applying and being in the program as an Indigenous person?”. This approach allowed students to share information most relevant to them without any influence, thus revealing the most salient aspects of their experience for them. The interviewer then used pre-developed prompts to ask follow-up questions to gain information on aspects the participant may not have shared, and to clarify information. The interviews were audio-recorded and then transcribed by a member of the research team (NM).

Data analysis was conducted by team members that represented both Indigenous and Western perspectives (CB, KH, NM, MF, GR, DB, LC). Data collection and analysis followed an iterative process. Following the first three interviews, members of the research team read through the interviews in full (CB, NM, MF, GR, DB, LC) and met with the interviewer (DP) to discuss emerging themes. We discussed how beyond the practical aspects of their stories such as, what went well for them in the program and recommendations for improvement, the participants primarily talked about Indigenous identity and how it related to their experiences as a health professional student. The team developed more probing questions for the interview guide (Appendix A) so that this theme of identity could be explored in more depth in the four remaining interviews.

All seven interviews were analyzed using codes that were determined following reading all seven interviews in full. The development of the codes was informed by a three dimensional space approach which involves analyzing the stories on three dimensions: (1) interaction of the participant with themselves (personal experience) and interaction with others, (2) continuity or temporality, where the transcript is analyzed with consideration for past experience, present experiences, and the future, and (3) situation or place in order to provide contextual physical information to the story [20]. Codes included: past context, profession discovery, applying to the program, being in the program, clinical practicums, and future plans. Once two researchers (CB, NM) coded the interviews independently using an excel spreadsheet for data organization, they met to compare the coding and resolved discrepancies with discussion. As the codes placed the interview content into chronological and contextual order, we then were able to develop a “mini-story” by stringing together the coded content from each individual together. In deciding which content to include or exclude for this paper, we focused on the content that specifically helped understand the student’s perspective on their identity. We ensured that we included content that was both similar to multiple participants, as well as unique to each participant. However, we included more content on aspects of the individual’s story that was unique as the variation in experience was a particularly striking finding. In developing the “mini-stories”, we repeatedly returned to the transcripts to ensure accurate representation of the participants’ experiences [26].

We emailed each participant their mini-story and asked them to provide feedback on whether the mini-story was representative of their experience. Two participants edited their story to make it more representative of their experience, three provided comments that resulted in changes, and the rest of the participants approved the story as presented to them. Once the mini-story was approved by the participant, it was minimally edited for length and grammar. Other strategies to ensure trustworthiness were coding and interpretation with team members that represented diversity in worldviews as described in our positionality statements. During knowledge translation of preliminary results in public forums, two participants approached CB and expressed interest in contributing to the next phases of interpretation and knowledge translation. They were added to the author team in the dual role of participant and researcher, and their input further shaped the presentation of the results and the points of emphasis in the manuscript development (DM, MC).

## Findings

Seven student participants that represented all three programs (occupational therapy, physical therapy, and respiratory therapy) within the studied rehabilitation program participated in this study. Their experience in the program ranged from first year students to students who had recently graduated and were employed as rehabilitation professionals. To protect the identity of participants we are using gender neutral pronouns and not specifying their professional program, unless they did not wish to be anonymous. The seven mini stories represent the participants’ experiences with Indigenous identity in learning about, entering and being in their rehabilitation educational program.

### Phoenix: “As an Indigenous person, I have a lot to talk about”

Phoenix is a member of a Manitoba First Nation that financially supported Phoenix’s university education. Phoenix believed in the importance of supports and resources for First Nations health professional students to increase the number of graduating First Nation health professionals. Phoenix thought that First Nations students would be more likely to return to work in rural communities, that they would relate better to the community, be more “sensitive to social issues” like systemic racism and be more understanding of cultural practices.

Phoenix received supports from university Indigenous resource programs prior to, and during, their rehabilitation degree. In their second year of university, prior to admission to the rehabilitation program, Phoenix was in an Undergraduate Indigenous Health Career Program designed to support Indigenous students interested in a health career. Phoenix felt this program was key to their success because they did not have enough positive social supports in their first year of university. In the Health Career Program, Phoenix found like-minded students and mentors that supported growth. From this program, Phoenix gained a sense of community and received tangible supports and resources like tutoring, counseling, cultural activities, small class sizes, and the opportunity to explore different health programs. The Health Career Program was instrumental in helping Phoenix explore a variety of health professions to find the best fit.

Phoenix felt they were thriving in the rehabilitation program because of support from their health professional classmate peer group. Phoenix saw parallels between their rehabilitation experience and being in the undergraduate Health Access Program, as the rehabilitation program was a community of like-minded individuals who were supported by mentors and instructors who were able to provide a personalized education experience. Phoenix was aware of Indigenous-specific supports they could continue to access once in the rehabilitation program, such as tutoring and counseling. However, Phoenix had not needed tutoring so far, and Indigenous-specific counseling was inaccessible as it was offered on a different campus. Phoenix’s identity was strongly related to being a part of their rehabilitation class. Phoenix’s experience is summarized when they say,

As soon as I got to class there, my classmates have been an endless stream of support. Um you know, just in terms of feeling comfortable on campus because you have people to talk to, you have people to bounce questions off of and ask about questions, or ask about an assignment, or double check when an assignment is due. You know when we have our group chat and everything, if you have any questions or need any form of support, uh you know, we just toss something out there. You know we had a guy just break up with his girlfriend, you know, and now we are all kind of, we coached him through that. So it’s just, and we’ve only known each other for two months, so it is just great to have that um, that social support. . . (I don’t miss the Undergraduate Indigenous Health Career Program supports) um other than that access to tutoring I find very valuable. Although I haven’t made use of it this year just due to like, my class having um, um, just due to my class being so supportive.

Phoenix was proud to be in the Indigenous admissions category, and proud of their Indigenous identity. Phoenix did not feel they were treated any differently as an Indigenous person. Phoenix said their classmates did not talk about cultural or spiritual identity with each other, but Phoenix had shared with some classmates that they were admitted in the Indigenous Peoples category and did not think this had changed the way their classmates treated them. Phoenix thought that other students worried that being in the Canadian Indigenous People category was stigmatizing and might be preventing them from accessing Indigenous-specific supports. Phoenix encouraged other Indigenous students to access the (Undergraduate Indigenous Health Career Program) services but didn’t think they ever did.

Phoenix doesn’t “really follow any cultural practices” but thinks that their First Nation connections help them see the world in a different and important way that is needed in the health professions. In talking about the admission interview, Phoenix said,

I found as an Indigenous person I have a lot to talk about, not just because I am Indigenous but because over one summer I took a job at a high school that was for Indigenous students that weren’t able to be educated via traditional means, like it they, like it was just for people who didn’t have access to high school so we had to have equivalency courses over the internet. So I just, I developed a really strong understanding of First Nations social issues especially related to health and education and I just felt I had a lot to talk about, due to that job but I know I wouldn’t have gotten that job if I wasn’t First Nations. . .even just being First Nations a lot of us have the um, an awareness of a lot of the social issues that are kind of going on right now, I’ve learned a lot about that at school. . .I felt that school in general as a First Nations student has just been, it’s been a lot easier to hone in on to social issues. And that gave me something to talk about when it came to… there was [one question during the admission process] that was talking a lot about social and societal issues and I, and that was just the best interview of my life.

Phoenix’s First Nations identity is a part of their being and motivation. Phoenix has a strong belief in the need for more First Nations health practitioners to serve First Nations people, regardless of their cultural practices. Phoenix seemed to have a sense of belonging with both their First Nation and health professional student identities.

### Franky: “I am proud to be in this category”

Franky is Métis and grew up in rural Manitoba. Franky said they are a minority in a couple of ways - in addition to their Indigenous identity, their first language is French. Franky thought that their rural community and schooling did “a pretty good job of introducing the cultures. . . but honestly not enough”. They felt this was contributing to the Métis losing their heritage because they are no longer connected to their roots and communities. Franky was grateful for the bursary and financial opportunities available to them as an Indigenous student; as a first-generation university student, this type of support was very important for their success. Similar to Phoenix, Franky did not access the Indigenous Health Professional Student Support program, saying that they found social supports in their rehabilitation program that were meeting their needs, but felt it was important for these resources to be available for Indigenous students that may require this type of support.

Franky felt that they were supported as an Indigenous person in the rehabilitation program in a few ways. One support was how the information on the Canadian Indigenous Peoples admissions category was embedded in the rehabilitation website, so that those already interested in the profession would see the information and perhaps think to themselves, “Oh this might be achievable for me.” Other things that were supportive of their identity were land and treaty acknowledgements by instructors, explicit advertising of the Indigenous Health Professional Student Support program, and the incorporation of culture and context into education materials. Franky specifically referred to case studies,

. . . that talk specifically about Indigenous Peoples and how approaching people, not even just Indigenous, but people from different cultures, and how social norms will vary and how cultures are very particular, and they make sure that we understand there are going to be different contexts as health practitioners.

Franky thought that working through these types of case studies were important for everyone to start to feel more comfortable with interacting with people with different cultures and identities and to reduce the pan-Indigenization of identity. Franky found these classroom case studies helped them feel more confident interacting with First Nations people on clinical placement.

Franky was proud to have been admitted in the Canadian Indigenous Peoples application category but discussed the potential negative view of others. Franky shared,

I feel like if other candidates that are also applying to this program might know that you applied, they might feel like you are just given this opportunity without having any academic strengths or personal strengths, like they were just maybe they were just admitted to this program because the (university) has a mission statement of acknowledging Indigenous um, status and culture, so I feel like there is maybe a negative connotation to that.

Franky spoke of the privilege they hold as someone who is not “visibly Indigenous”, and being able to choose if they will share their Indigenous identity with others. Franky sometimes self-identified themselves to clients that were also Indigenous while on clinical practicum, and when they did, “it’s easier to create rapport for sure”. This was “because of similar cultural practices that you can just like discuss and share with patients”.

Similar to Phoenix, while Franky considers their Indigenous identity as a part of their being, they were very focused on the development of their professional identity and found belonging in their choice of profession and classmates. Franky spoke about what needs to be done for Indigenous students as a whole rather than themselves and their own identity. Franky is a strong advocate for improving curriculum and education on Indigenous issues, health, and culture across the education continuum, from elementary school through to university level.

### Corrine: “I needed to learn to fit in”

Corrine is a band member of a Manitoba First Nation and lived on reserve until the age of 17. At the time of the interview, Corrine was struggling in her rehabilitation program, and linked this directly to her First Nation identity. Corrine had a history of trauma and racism in her childhood and undergraduate experiences. She said: “As a result of the trauma my whole life, I haven’t been able to function as well as I could”. Just considering a health profession as an option was difficult, because of the competitive admissions processes of the programs. Corrine said she carried internalized racism, always feeling “not good enough” even once she was in the rehabilitation program. Fears of stigma related to being admitted in the Canadian Indigenous People’s admission category brought up feelings of self-doubt: “I still feel like, almost like ashamed sometimes. I wouldn’t say ashamed but like I get scared because of that I feel like I am still not good enough”.

Corrine said that the way the curriculum was taught in her rehabilitation program was not supportive of her identity. She said:

It’s hard because it’s a very Western, well it’s a very for blunt terms it’s a very a white program. The curriculum is very white, and I think what really sucks, in my opinion, I always struggle with it, being the obviously Indigenous looking [person]. I feel like people don’t notice it -people won’t acknowledge, but you can feel that they see you as less than, because you are Indigenous. I hate it because whenever something Indigenous is brought up in the courses then it’s like people like don’t know how to react. I have spoken up a few times about the culture, but I don’t know if that’s impacted people, and the hardest thing, like the hardest thing that I had to go through was I have to change parts of me just to succeed in the program.

She said there were a “few times” where there was Indigenous-oriented content but that it was insufficient, and “it really shocked me when we did a sharing circle one time, and then there were people didn’t even, this was their first time learning about residential schools. . . it’s just opened my eyes to just how blind people are still to the history that has happened to our people and you know the stigmas, like things like that.”

Corrine had a traumatic experience in a clinical placement, where she felt she was labeled as non-approachable and was asked to change the way she carries herself. This was difficult to take when she already felt like she was working to ‘conform’ and was devastated that she still needed to try to change who she was to succeed in the health care professional program. At the same time, she was also witnessing racism in this clinical placement but found that “people won’t notice it -people won’t see it”. The layering of racism in the clinical setting resulted in Corrine not completing her first placement and needing to take time for herself to rebuild her identity and confidence. Corrine was forced to reflect on her past, her family’s history and how this affected her as she worked to find a way forward in her health professional program.

I, just, it was just hard I didn’t grow up to be someone who would just go in and be like hey!, and stuff like that. It was almost like, like we grew up to wait for people to talk to us, and I think that’s an outcome of the residential school system, because you couldn’t speak until you were talked to, or you’re told to do, and I think that’s how I grew up and that’s seeping into here now, because I can’t do that, and when I first started trying to do the eye contact thing, it was traumatizing, it was extremely traumatizing for me and I had to fight to learn how to do that myself.

Corrine’s identity and past also made it difficult to feel connected to the other students in the class. She felt like she did not have enough in common to be able to talk to them.

It’s also just very hard to talk to people because I can’t relate to them on the same level, they talk about things that are just, in their personal lives and for me I didn’t grow up talking all the time, you know like, it just, it’s hard, I can’t relate to people.

She provided an example of how interacting with other university students is difficult because of double standards that exist because of racism. “Our people get called drunks and it’s so negative, but then on the other side it’s all, ‘oh let’s party, let’s get drunk’, and it’s just like, I can’t stand that”.

Corrine felt compelled to educate others about racism and First Nations people and made herself vulnerable to her classmates by being a voice for First Nations people in the classroom. Thus, while in the rehabilitation program, Corrine had a large additional emotional load beyond developing her professional identity. She was continually shielding her cultural identity from harm, reprocessing her past and present, and working to negotiate her identity and how to present herself in the Western educational and health system and to her peers, without the benefit of strong social supports within the classroom.

Despite all the challenges to her Indigenous identity in the program, Corrine’s strong First Nations identity drove her to be persistent with her goal to be a rehabilitation professional. She had learned about her chosen profession when she was in high school, when her family required health care services. She saw becoming a rehabilitation professional as an important way to give back to her community. “I really am in this program to make a difference …, to work with the people up north, because I know there is a lack of sensitivity to the culture, sensitivity to the history of them, of our people.” To promote her success, Corrine accepted support from some faculty with whom she felt safe, and the Indigenous Health Professional Student Support program. While Corrine was working very hard to succeed within the system, she was resentful that she needed to conform to the “white man’s world”, a place where she felt she would not be able to succeed. While Corrine was strongly rooted in her First Nations identity, she struggled with developing her rehabilitation professional identity because of the lack of continuity between her First Nations identity and the Eurocentric rehabilitation education environments.

### Carrie: “It’s important to show that Indigenous people are here”

Carrie is Métis with a European mother and a First Nations father. Carrie applied in the Canadian Indigenous Peoples category because,

I always self-declare. It’s important to know that more Indigenous students are going to higher levels of education and I think that maybe because I want to learn more about that part of my culture and then if I put it out there then it just makes it something that I continue to look into and follow up with. . .For me, I want to represent the category. I get tired of hearing of people say things like ‘oh well hasn’t it just been long enough and shouldn’t they just figure it out’, and so for me by self-declaring and choosing to be open about being a part of that category it’s saying that there are people who are going to school and it may be slow but it is happening um, you know and when people say things like that I just wanted them to open their eyes a little bit more.

Despite this confidence in self-declaring at the time of application, at the time of the interview, they were having some questions around “the purpose of self-declaring and what does it mean?” Carrie heard of other students who did not self-declare because they did not feel any financial or social need for being identified as Indigenous at an administrative level and wanted to avoid stigma from other students who saw the Canadian Indigenous Peoples category as an opportunity to get into the program with a lower GPA. Since Carrie had been accepted to another highly competitive university in the past, they were able to reflect on these different perspectives without taking it personally, but it still took cognitive energy.

Carrie shared how cultural identity has been a lifetime struggle for them:

I always struggled with where to identify because growing up I know my dad was having, like he had a hard time keeping a job because he would come home and talk about like somebody saying something racist to him, and then he would just wouldn’t take it and then he would end up getting fired from his job. So then I identified with my dad experiencing those racist like comments and experiences and I grew up going to pow wow dancing, when we were kids we did that every week with my cousins and family members, my aunts and uncles and everybody would go. So I identify with that part of my culture. And then I always felt like I didn’t know how to pursue it more on my own or to feel comfortable in those environments where people are discussing the pain that they have had, or their experiences or what they know about their culture because I always felt like I didn’t look, I never felt like I was accepted. I always think in my head it’s because I don’t look Indigenous enough or just because of the way that I came out [looking more like my mother]. How I experience it is I want to be- want to advocate for better health care for First Nations people but I feel like I don’t know where I fit into.

Carrie sought out the Indigenous Student Health Professional Support Program which they found to be an important support, providing a cultural social space and opportunities to explore their Indigenous identity. Carrie had some tension in relation to sharing with other students about their use of these supports because non-Indigenous students had difficulty understanding the cultural support component of the program. Rather, the non-Indigenous students focused on the pragmatic supports it provided. Carrie didn’t want to have to be apologetic about the services they sought at the Indigenous Health Professional Student Support program and wished non-Indigenous students could be more informed.

I know some of the other students (at the Indigenous Student Health Professional Support Program), in the (another anonymized health professional) program especially, some of the stories that those students would share when I talked to them. . . I just thought like wow if you are just dealing with some of those microaggressions all of the time, and some of them weren’t micro even, um you know, just having free printing isn’t making up for it, you’re not really getting like a step up, it’s just a small thing that’s helpful.

In addition to trying to be a positive role model themselves, Carrie thought that it was important for the rehabilitation programs to increase the positive representation of Indigenous Peoples in the program. They provided examples such as including positive characteristics of Indigenous Peoples in case studies and bringing in more positive Indigenous role models into the classroom, like Indigenous Elders. Carrie was proud of their Indigenous identity but was still looking for belonging. Carrie seemed to be developing their Indigenous and professional identity in tandem while in the rehabilitation program.

### Pat: “I kept it quiet”

Pat is Métis. Pat applied to their rehabilitation program at the same time as a friend who was also Métis which provided Pat with social support throughout the admission process. Pat was very thankful for the Canadian Indigenous Peoples category because their GPA was likely not strong enough to get into the program through other admission categories. However, this created a situation where Pat was hesitant to share their Indigenous identity with classmates.

I kept it quiet, um, not that I didn’t want people, or not that I cared if people knew but I kind of didn’t want to be labeled into this category of ‘oh you only got in because you are in the category’. Um and possibly the sense of ‘our GPAs are better’ I guess was kind of at the back of my head. But I think overall nobody ever demonstrated that behaviour, so it was maybe something I was thinking personally, but I felt safe the whole time if that makes sense.

Very slowly over time Pat started to reveal their Indigenous identity within their rehabilitation program. In their second year of a two-year program, they learned about the Indigenous Health Professional Student Support program and started accessing it with their friend who had also been accepted into the program.

At the beginning I was a little bit more, I didn’t really want to get involved too much, and again that might have been an identity thing and I just kind of wanted to stay in the background. But because of my friend who was willing to put herself out there I’d get involved (in the Indigenous Student Health Professional Support program).

The tension Pat felt with “putting themselves out there” continued until graduation. They declined the graduation Pow Wow Ceremony. But at the cap and gown ceremony, they decided at the last minute to wear the purple sash that identifies Indigenous graduating students.

I didn’t want to stand out as different from everybody else. . . and I didn’t want to put myself out there and then when it actually came to grad day and my friend was doing it, it was like ‘you know what, NO I am doing this, this is who I am’, um, so I ended up I guess putting that identity out there.

In retrospect, Pat had some regret that they did not take up a lot of the opportunities that were available in the Indigenous Student Health Professional Support program to learn more about their own cultural identity.

I think because I shied away from it so much at the beginning because I didn’t want to be labeled as different. If I would have known that you know in the end there’s really, it doesn’t matter where you come from or who you are, everybody’s treated the same (at the Indigenous Student Health Professional Support Program). It would have been nice to have access to everything and put myself into the different programs that were going on or go to the events.

Through the interview, Pat spoke little of their understanding of their Indigenous identity and history. While they recognized they are Métis, they seemed to have limited personal understanding of their identity. During their health professional program, more curiosity emerged about it, and when they were ready to address this part of themselves, their journey through the health professional program was over.

### Melissa: The application process stimulated a self-discovery journey

Melissa is Métis and grew up in a rural community. Melissa always knew that her family was different, but it wasn’t until university that she was “able to have an identity”. As she prepared to apply to her preferred rehabilitation program, she noticed that there was a Canadian Indigenous Peoples admission category. This prompted her to learn more about her own history. Melissa explains,

It didn’t happen until this (rehabilitation) program where I really started thinking, and expressing myself as a Métis student, or a Métis practitioner now. I didn’t put a label on myself and so then my lens of being an Indigenous learner didn’t happen until I came into this program, because it was kind of like a transition point in my life where I was going through the process of getting my Métis card, but also talking to my family about it. It was never really brought up in my family before that point, but now I understand why my grandpa speaks another language, and I never knew what that language was, and it’s Michif.

Melissa was grateful to have been admitted in the Canadian Indigenous Peoples category. “It’s nice to have some identity where I didn’t before and for various reasons my family didn’t talk about it, and so now (the admission category) opened up my opportunities with exposure to this program (the Indigenous Student Health Professional Support Program).” However, Melissa did sometimes ask herself “do I belong?” (in this admission category).

I think that’s kind of the question with Métis a lot, because we are stuck, it’s two different worlds that we kind of got a foot in, and I think that’s where I struggle, like ‘am I Indigenous enough to be here?’. . .but that quickly squashed when I actually came here (to the Indigenous Student Health Professional Support program) and I got to learn so much about myself and Indigenous culture because of that. . . it was really helpful because at the time I was learning about my cultural identity, my past, everything like that was all at the same time and it helped having this safe place to learn all that stuff.

During the rehabilitation program, she continued to develop her identity as Métis and her understanding of how this part of her identity influences her work as a rehabilitation professional.

I think that’s where (during the program) I started asking the questions ‘What does it mean to be Métis?’, ‘What kinds of experiences do Métis people have in the health care system?’ and ‘What can my role be, being a Métis practitioner?’. I think that’s been in my mind throughout the entire time – okay once I graduate what am I going to do for my community, what am I going to do here?

This reflection on her identity and how it related to her career continued into her clinical practicums. Melissa was learning to see the world and her role in it in a different way. For example, in talking about her experiences in the health care system before being in the rehabilitation program, compared to now, she said,

I don’t look Indigenous, I don’t look the typical part, and I didn’t know I was Métis in any way growing up. I didn’t know what that meant for me really, so for me I guess in my past, it hasn’t affected me. Now that I have a different lens opening things up, now I see health care differently. When people ask me questions, I think, ‘oh, would you ask this to someone else, maybe who’s different skin colour?’ Like I am always kind of thinking about that, but for myself I don’t think being Métis had a significant affect on my health care experiences because I could hide it. And that’s the unfortunate part.

Melissa noticed the over representation of First Nations, Métis, and Inuit people as care recipients in her clinical practicums, which took place in an addictions program and a mental health program. It prompted reflection on what it meant for her as an Indigenous rehabilitation professional. “That over representation was at the forefront of my mind all the time and thinking - what can I do as a Métis, a future Métis practitioner - what can I do to help, and what kind of role can I have in this.”

**Fig. 1:**
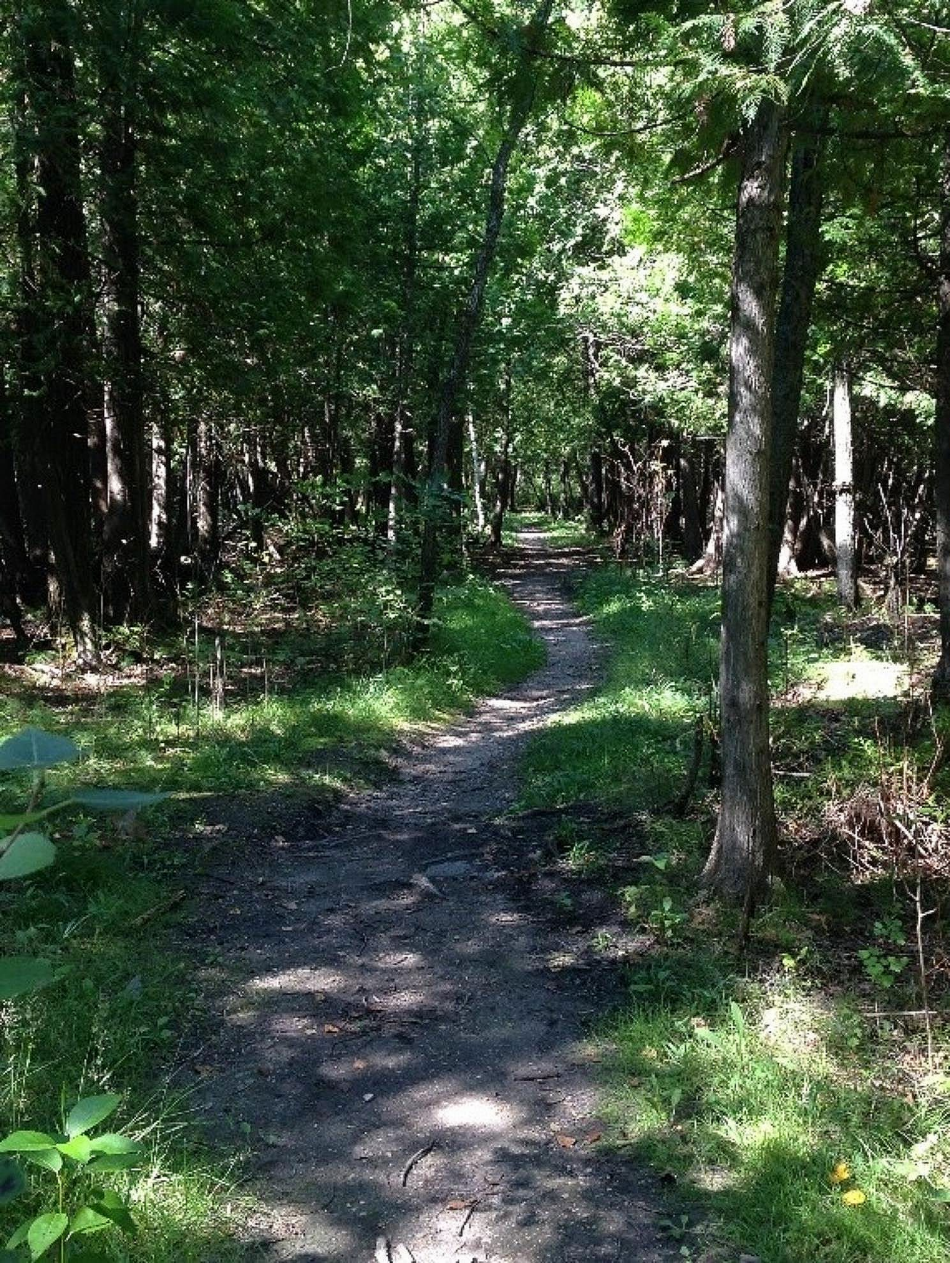
Melissa’s identity journey. This photo was taken by Melissa and she feels it represents the winding identity journey she began when admitted to the Indigenous admissions category and that continues today. The visible roots symbolize the family history she is stumbling on and appreciating while reclaiming what was lost

Admission to the Indigenous Peoples category prompted what Melissa described as a “winding identity journey” (See Fig. 1). Her professional and cultural identity were both born and developed in tandem through her journey of getting into and being in, the rehabilitation program. Being in the Canadian Indigenous Peoples admission category drove her to consider “what it means to be a Métis practitioner”, and to develop this identity throughout the rehabilitation program. For her, the opportunities facilitated by the Canadian Indigenous Peoples category opened simultaneous doors to a health care career and finding hers and her family’s identity.

### Dustin: “It’s not important that I am Indigenous”

Dustin grew up on a First Nations reserve in Manitoba until grade 10. Dustin did not learn about the Canadian Indigenous Peoples admission category until he had already decided to apply to the program and was surprised that there were reserved seats for Canadian Indigenous Peoples. Dustin did not experience any difficulties in the program admission process and did not think there were barriers for Indigenous Peoples in the process. He found the workload high but found ways to manage it. He found the instructors to be supportive and helpful.

Dustin signed up for the Indigenous Student Health Professional Support Program but did not use the services or the space as he preferred to study with his classmates.

I signed up here (Indigenous Student Health Professional Support Program), but I had no time to be here, and I just didn’t find it helpful to be here, cause everyone else in the program is studying over there, so I may as well study with them. But I am not going to come over here and study by myself right, and I just didn’t really find it necessary or useful to come here.

However, he thought that these services might be important for some Indigenous students.

When asked about being able to maintain his cultural identity in the program, he said,

I don’t think it’s important that I am Aboriginal. . . it’s just that I’d rather be thought of as a person -it’s kind of how I approach things, like culture identity. Like, I am not ashamed of it, it’s just I’d rather just be looked at as another person. I guess it’s fine if you were to do it, I don’t think it would be a problem, but for me personally, I don’t think it’s always necessary to do that. . I feel pretty comfortable with who I am in society I should say, I guess certain people from different situations can kind of, or different backgrounds as being an Aboriginal person, probably have a difficulty fitting in, but personally I haven’t really found any barriers or anything like that.

Dustin felt there were no barriers to his journey into and through his rehabilitation program related to his Indigenous identity. He felt grounded in his cultural identity and did not feel the need to explore or develop this part of his identity in the program. He did not speak of any incongruencies or challenges between his values and the program (both curriculum and clinical practicum) and he was focused on developing his rehabilitation professional skills with his peers.

## Discussion

This paper provides insight into the experiences of identity of seven Indigenous rehabilitation students. These students are all now therapists working in their chosen profession. This is a unique contribution to the literature as we know of only one other peer-reviewed study that examined Indigenous identity in health professional students and it did not include rehabilitation students. Previous works have already highlighted how Indigenous students are a diverse group of people (rural, remote, urban and from different Indigenous nations); in those studies, the student experiences were combined in the findings [9, 11, 27]. The students in this study spoke of some aspects of being an Indigenous post-secondary student that have been discussed in other literature, such as how Indigenous students have a more collective motivation for schooling (e.g., giving back to their community) and how they experience racism in their everyday lives [11, 13, 14, 28, 29]. Our study provides more insight into the identity diversity within Indigenous health professional students by presenting their seven unique stories individually.

Our study is particularly important for highlighting diversity in the dual development of Indigenous and health professional identity. The only other study we found that focused specifically on Indigenous health professional identity development was a mixed methods PhD dissertation by Penfold, an Indigenous psychologist. She also found that Indigenous identity was highly variable between students [27], and her quantitative data suggested that variation depended on several factors, including whether the students were Métis or First Nations, and when they found out about their Indigenous identity. Penfold used a social identity theory by Roccas and Brewer [30] to explain the identity patterns in her participants. Some students compartmentalized their identity, expressing their Indigenous identity in some contexts and situations but not others (i.e., not in the educational setting). Penfold also had some participants who merged their identities – in this case, they were the whole of all their identities. For example, in our study, Melissa who was seeking to develop herself into a Métis occupational therapy practitioner, developed her professional and Indigenous identity as one. Another way to consider variation in self-identification is to consider the three stages of identification discussed by Coates [31]. First, an individual needs to self-identify – to see themselves as Indigenous. For example, Pat, in our study. Second, the individual connects to their own cultural or political group, such as Melissa in our study. Third, the individual identifies with additional Indigenous groups and cultures across Turtle Island. This could be the situation for Dustin, who was very grounded in his identity, and focused on developing his professional identity. The development of Indigenous professional identity is an area for further exploration.

The student stories presented in this paper provide insight to health professional educators on the individual nature and complexity of Indigenous student identity. Having this understanding is important because one of the most significant facilitators for Indigenous student success is social supports, including high quality instructor-student relationships [32]. A deeper understanding of Indigenous student diversity is important to ensure that instructors are not considering Indigenous students with a pan-Indigenous lens. Failing to see Indigenous identity as varied, and unique to the individual can impede or harm instructor-student relationships. However, there also needs to be structural changes to health professional educational institutions to reduce cultural incongruity for Indigenous students. Some students, like Corrine in our study, are put in the difficult position of trying to adopt some aspects of professional identity that are in opposition to their cultural identity. The experience of cultural incongruity with professional identity can put additional strain on students as they try to navigate how to reconcile the incongruency [27]. Students who experience discontinuity between their cultural identity and the health professional culture to which they want to belong can experience additional challenges and stressors in already academically intense health professional programs [27].

To reduce incongruency, changes need to be made at a structural level in curriculum and educational culture, and not just be ‘add-ons’. Many Indigenous scholars have previously proposed structural university transformation to create an environment for Indigenous students to thrive. Pidgeon [28] envisions that “a truly decolonized institution would place Indigeneity at its centre; how the academic governs, discusses and enacts would be inclusive of Indigenous ways of knowing and being” (p. 33). In this way, Indigenous students would be in an environment where the learning helps them better understand themselves, their families, and communities in a way that is healing and provides them a platform for their journey after university [13]. Chenoweth [33], an Okanogan band member (the silyx people) sees identity as a core element for Indigenous Peoples in institutional university transformation. He re-imagines university education for Indigenous Peoples and communities by using Indigenous worldviews for rebuilding “educational models by and for Indigenous people” (p. 34) that addresses Indigenous community needs in relation Indigenous epistemologies and language revitalization.

Steinman and Sanchez [34] emphasize that the process of Indigenization – meaningful changes to practices and processes in universities to include Indigenous Peoples, and ways of knowing and doing things - must be done thoughtfully to minimize the re-creation of harm. If universities prioritize public and explicit changes, for example, celebrating the hiring of Indigenous faculty and incorporating Indigenous art into the environment, without deconstructing policy that upholds colonial power structures, this contradiction can perpetuate colonial power dynamics and harm to Indigenous Peoples. This is an important lens in considering how to reduce cultural incongruity in university settings.

The students in this study identified several important ways that cultural incongruity can be reduced that is supported by literature and Indigenous leaders. The most important of these is hiring Indigenous faculty staff, and support services and ensuring they are included meaningfully in all aspects of the academic institution [34, 35]. Indigenization in the university is not possible without Indigenous leadership and working partnership between Indigenous and non-Indigenous faculty and staff. A critical ingredient in the transformation at our institution has been the creation of Ongomiizwin – Indigenous Institute of Health and Healing. Its mandate is to provide leadership and advance excellence in research, education and health [36]. While our rehabilitation programs are currently working towards increasing the number of Indigenous staff and faculty, we have fostered a sustained relationship with this institute to advance our goals related to Indigenization initiatives in admissions, creating safe learning environments, student support, and linking to Indigenous communities for reciprocal working relationships. Ongomiizwin staff, Elders and Knowledge Keepers have been included in our decision-making settings and in celebrations to support our capacity to advance the Truth and Reconciliation Calls to Action [37].

The participants in this study also identified that a relationship approach was particularly important for achieving their academic goals [32, 38]. The participants identified small class sizes, the opportunity to develop relationships with Indigenous and non-Indigenous students, feeling supported by faculty, and having Indigenous student support services available to them as important positive aspects of their academic experience. To consider how a relational approach can be embedded in the structural environment, instructors and leaders can use the *Cultural safety and trauma- and violence-informed care framework for redressing inequities in healthcare access* [39]. This framework has three primary domains for development. First, it guides the development of reflexivity to make oneself more aware of their own power, privilege, values and beliefs and how this impacts their relationships. Second, it provides guidance for how to prioritize relationships, critically evaluate one’s own approach to relationships, and develop trusting and supportive relationships. Third, the framework promotes consideration of context and thinking around how to interrupt structural barriers. This framework can support a relational approach with students that will help them be supported in a way that honours each of their unique identities, since the most powerful findings of this study was the inherent variability in the Indigenous identities and their individual needs and preferences. It can also help educators consider the broader context of students for reducing cultural incongruity, such as supporting the examination of both explicit and “hidden” curricula and the messaging that is being delivered to students regarding health professional identity and belonging.

The participants in this study greatly appreciated the option of having Indigenous services, supports and spaces in both their undergraduate and health professional degrees. Unfortunately, a common situation can be that these services are provided as “add-ons”. Some authors point out that this social construction mirrors how Indigenous communities have been forced into the peripheries of Canada [14, 33]. Spaces that provide an environment of safety for students are very important and the Indigenous student space was an important support and a place for cultural growth for some of the participants in this study. However, some participants felt that spending time in the Indigenous student space took away important time with peers in their programs. Building social capital is important when working to be accepted in a new social group, and the building of this social capital requires spending time in the learning environment [9]. Health professional programs have intense schedules with limited free time. Thus, students need to make choices about where they want to spend their time building social capital. With structural transformation that is inclusive of Indigenous ways of being and knowing, students would not be put in the position of needing to make these choices between Indigenous and non-Indigenous spaces and may be more empowered to develop their identity as an Indigenous health professional.

This study advances knowledge related to student experience in relation to being admitted to a heath professional program in an Indigenous Peoples priority category. Our study confirms findings by Cox-White and Penfold [11, 27] that students experience and worry about experiencing stigma from other students because of an alternate admission pathway. Our findings expand on how students can be concerned about the perpetuation of stigma and racism because there is not enough understanding of the purpose of Indigenous student admission categories and the resilience of Indigenous students at the post-secondary level. This means that for some students, the stakes for stepping forward as an Indigenous student can feel high when also working to develop a health professional identity with peers [13]. However, the students in this study all felt that this admission category was important for a variety of reasons, including reinforcing identity and providing opportunities for Indigenous students. While we cannot know all of the reasons for students to want or not want to share or focus on their Indigenous identity, it cannot be ignored that the historical and present stigma related to being Indigenous is a powerful factor in Indigenous identity sharing, and that this particular reason for not wanting to share or express their identity needs to be eliminated in university education contexts. As Weaver stated [15], context influences whether someone keeps their identity quiet or chooses to use identification as a tool in resisting assimilation.

There are several important research directions on Indigenous student identity and health professional education. Since different universities are in different phases of Indigenization, it would be helpful for these processes and associated evaluatiosn to be shared, so that universities can learn from each other. This would include hearing from both Indigenous and non-Indigenous health professional students post-graduation, to hear about how the educational experiences have and have not helped them develop their identities and prepared them for working with Indigenous Peoples and communities. It also is important to integrate Indigenous community voices into this topic, to learn more about how universities can respond to community needs in relation to the education of Indigenous and non-Indigenous rehabilitation health professionals to support community health and wellness. Since this study and another unpublished study [27] have identified a high level of variation in the identity of Indigenous health professionals, developing more understanding on how to ensure that universities are able to support all these unique needs is an area for further exploration.

The voices of Indigenous rehabilitation students are important to listen and respond to because there are currently very few Indigenous rehabilitation professionals. These voices have important roles in transforming how rehabilitation university programs are developed and delivered for Indigenous Peoples, and how rehabilitation services are provided to Indigenous Peoples to promote culturally relevant care and healing. While being an Indigenous student in a colonized university environment puts more pressure on Indigenous students as they work to reconcile their own worldview with that of the colonizing university environment, the university setting can play an important role in supporting personal healing and empowerment of Indigenous Peoples [9, 13]. This environment is where Indigenous students can learn more about the history of colonization, and the resiliency of the generations before them, and how this contributes to the identity that they hold, as was seen with participants in our study such as Corrine, Pat and Melissa [13, 40]. It is imperative to support these students, who Drywater-Whitekiller [40] deemed to be “modern day academic warriors” (p. 3), to develop into professionals who bring their full self to their professions.

A strength of our study is that we verified the findings with the participants and invited participants to be a part of the data analysis and knowledge translation team using a relational approach [41]. All participants were included in developing their story and main theme (as represented in their story title); three participants wrote or edited their own story as presented in this article. We centered Indigenous authors and their perspectives in the discussion of the paper and in deciding the main messages to be shared. Our team attempted to de-emphasize the colonial lens in exploring these students’ experiences of identity by providing a first-person account. We had Indigenous team members in all stages of the research process, but the team lead is a white settler with Western graduate education. Therefore, our research process was informed by qualitative methodology. The use of Indigenous research methods with an Indigenous scholar as principal investigator would have produced different results that may have allowed for more insight into this topic with an Indigenous worldview. Another limitation is that this study provides only a snapshot in time which is a limited method for looking at identity, as identity is dynamic over time.

In conclusion, these seven students generously shared their voices to help us understand the variability of identity experiences of Indigenous rehabilitation students. As academics and educators, it is our role to listen, reflect and work with Indigenous students to enact change in university settings. The responsibility to highlight the barriers to student success and advocate for change cannot be a burden left to these students [42]. Rather, we need to consider their voices as we collectively work towards action. Indigenous Canadian scholars have strongly stated that this action needs to include pervasive integration of Indigenous ways of knowing and being throughout university campuses, not just in corners of them [33]. Supporting the unique Indigenous identities in rehabilitation professional students is an important tool to support the development and implementation rehabilitation health services that are grounded in Indigenous culture and rights.

### Electronic supplementary material

Below is the link to the electronic supplementary material.

Supplementary Material 1

## Data Availability

The datasets generated and/or analysed during the current study are not publicly available to protect the participant’s confidentiality, but data at an aggregated level are available from the corresponding author on reasonable request.
