# Supplementary material for: Diversity and development of Indigenous rehabilitation professional student identity
Source: BMC Med Educ. 2024 May 30;24:595. doi: 10.1186/s12909-024-05576-y (PMC11138084; doi:10.1186/s12909-024-05576-y)
Supplement: Supplementary file 1 — Supplementary Material 1 [file 12909_2024_5576_MOESM1_ESM.docx]

# Appendix: Interview Guide

## Introductions

## Main Interview Questions

- What professional program are you in? Occupational Therapy, Physical Therapy or Respiratory Therapy?
- What year are you in the program, or when did you graduate?
- Tell me your story of how you learned about *(occupational therapy, physical therapy OR respiratory therapy*), your experience with applying to the program and your experience of being in the program as an Indigenous person.
- *At end of their story*: Thank you, that is really helpful. Is there anything else you would like to share about coming to, and being in the program as an Indigenous person?

## Prompt Ideas for Different Aspects of the Story

Use these prompts to help the participant discuss all aspects of their story.

### Where I came from:

- What has been your experience as an Indigenous learner?
- What has your experience been as an Indigenous person in the health care system?

### Recruitment/Getting ready to apply:

- How did you decide that you wanted to be (*occupational therapist, physical therapist, respiratory therapist*)?
- How did you come to learn or know about (*occupational therapist, physical therapist, respiratory therapist*)?
- Was it easy to find out about the program here at the university and how to apply?
- What was your experience with completing the application requirements, like the pre-requisite courses?

### Admissions

- Why did you choose to apply to the program in the Canadian Indigenous Category/How do you feel about being in this category?
- What are the pros and cons to being in this category?
- What was your experience with trying to get into the program?
- What was your experience with the admission interview or the MMI as an Indigenous person?
- How you think we could get more Indigenous applicants to your program or other programs in the college of rehabilitation sciences?

### In the program

- As an Indigenous person, did you/do you feel safe in the program?
- As an Indigenous person, do you have any suggestions or critique of the curriculum content?
- As an Indigenous person, what was your experience in fieldwork?
- What things have helped you be successful in the program?
- What things have been challenges for you in the program?
- Have you been able to maintain your cultural identity while in the program?
